# Should We Void Lactate in the Pathophysiology of Delayed Onset Muscle Soreness? Not So Fast! Let’s See a Neurocentric View!

**DOI:** 10.3390/metabo12090857

**Published:** 2022-09-13

**Authors:** Balázs Sonkodi

**Affiliations:** Department of Health Sciences and Sport Medicine, Hungarian University of Sports Science, 1123 Budapest, Hungary; bsonkodi@gmail.com

**Keywords:** delayed onset muscle soreness, lactate, bradykinin, Piezo2 ion cannel, proprioception, muscle spindle

## Abstract

The pathophysiology of delayed onset muscle soreness is not entirely known. It seems to be a simple, exercise-induced delayed pain condition, but has remained a mystery for over 120 years. The buildup of lactic acid used to be blamed for muscle fatigue and delayed onset muscle soreness; however, studies in the 1980s largely refuted the role of lactate in delayed onset muscle soreness. Regardless, this belief is widely held even today, not only in the general public, but within the medical and scientific community as well. Current opinion is highlighting lactate’s role in delayed onset muscle soreness, if neural dimension and neuro-energetics are not overlooked. By doing so, lactate seems to have an essential role in the initiation of the primary damage phase of delayed onset muscle soreness within the intrafusal space. Unaccustomed or strenuous eccentric contractions are suggested to facilitate lactate nourishment of proprioceptive sensory neurons in the muscle spindle under hyperexcitation. However, excessive acidosis and lactate could eventually contribute to impaired proprioception and increased nociception under pathological condition. Furthermore, lactate could also contribute to the secondary damage phase of delayed onset muscle soreness in the extrafusal space, primarily by potentiating the role of bradykinin. After all, neural interpretation may help us to dispel a 40-year-old controversy about lactate’s role in the pathophysiology of delayed onset muscle soreness.

## 1. Introduction

The pathophysiology of delayed onset muscle soreness (DOMS) is not entirely known, and has remained a mystery for over 120 years. It used to be a popular notion that the buildup of lactic acid could be blamed for muscle fatigue and delayed onset muscle soreness. This belief is widely held even today, not only in the general public, but within the medical and scientific community as well. However, studies in the 1980s largely discredited the role of lactate in DOMS [1]. 

Unaccustomed and strenuous exercise entailing repetitive, fatiguing eccentric contractions often induces DOMS. DOMS is defined as delayed onset of soreness, muscle stiffness, swelling, loss of force-generating capacity, reduced joint range of motion, and diminished proprioceptive function [2]. The pain of DOMS evolves in about 8 h, peaks 1 or 2 days later [3], and subsides in 7 days [4]. 

Several pathophysiological hypotheses have been proposed in order to explain the mechanism of DOMS. These include lactic acid, muscle spasm, inflammation, connective tissue damage, muscle damage, and enzyme efflux [5]. A new hypothesis put forward is that DOMS is an acute compression proprioceptive axonopathy, and could be caused by mechano-energetic microdamage of the proprioceptive terminals in the muscle spindles, due to cognitive demand-derived acute stress response (ASR) on top of unaccustomed or strenuous repetitive eccentric contractions [6]. It is noteworthy that research in support of the neural microdamage and resultant neuroinflammation theory of DOMS [7,8] has been on the rise. Current opinion highlights the potential role of altered lactate metabolism in DOMS, if the neural dimension is not overlooked and factored into the pathophysiology. 

## 2. Lactic Acid Theory of DOMS

It was a widely accepted scientific theory until the early 1980s that elevated lactic acid was the cause of DOMS. It was viewed that the accumulation of toxic metabolic by-products exerts noxious stimuli and delayed onset of soreness [5,9,10,11]. Indeed, lactic acid increases in muscles and blood right after exercise [1]. However, the lactic acid theory of DOMS was largely refuted based on the scientific observation that concentric (shortening) exercise is accompanied by higher metabolism and lack of DOMS [1,12], in contrast to eccentric (forced lengthening) exercise [12,13]. Furthermore, lactic acid levels return to pre-exercise levels within an hour after exercise and the timeline of blood lactate levels do not correlate with the soreness timeline in DOMS, regardless of level or downhill running [1]. Furthermore, this study showed significantly elevated lactic acid concentration during running on the level, but no elevation in downhill runners [1]. Léger et al. concluded, based on their study, that lactic acid cannot cause delayed onset of soreness, but may contribute to acute muscle soreness after intense exercise, due to fatigue [4,5,14].

## 3. Eccentric Contractions and the Injury Mechanism of DOMS

It is important to understand the type of contraction and the resultant injury mechanism that could lead to DOMS in order to understand the metabolic role of lactate in the proprioceptive terminals of the muscle spindle.

### 3.1. Neuro-Energetic Aspects of Eccentric Contractions

Unaccustomed and strenuous eccentric exercise often causes DOMS. Eccentric or forced lengthening contractions are guided by Type Ia and Type II proprioceptive neurons in the muscle spindle. It is important to note that the neural control of eccentric contractions is considerably different from concentric and isometric contractions [13,15,16]. Eccentric contractions have lower motor unit discharge with higher force generation [15,16,17] and energetic profile [18] than concentric contractions [13]. However, this positive energetic profile of eccentric contractions likely comes at the price of increased proprioception and a resultant heavier load on the sensory afferent terminals of the muscle spindle [19]. For example, downhill running with forced eccentric or lengthening contractions demands more proprioception and postural control, meaning heavier loading on the proprioceptive afferents in the muscle spindles. It is noteworthy that the proposed ion channel that could be involved in the proprioceptive terminal microinjury of the acute compression axonopathy theory of DOMS is the Piezo2 [7,20,21,22], and indeed, Piezo2 is shown to be the principal mechanotransduction channel for proprioception [23].

One neural feature of eccentric contractions is that higher cortical excitation and lower motor unit discharge is used [13,24]. Moreover, eccentric contractions have the ability to absorb energy from an external load [18], support the body against gravity, absorb shock, and store recoil energy for accelerating contractions [13,25]. Due to these characteristics, eccentric contractions were coined as negative muscular work by Abbott et al. [18]. Nonetheless, it has been noted that there is no such thing as negative work, according to physics [19]. Rather, eccentric contractions have the capability to store recoil energy that could come from, e.g., ground reaction forces [19]. The problem is set to arise when the storing of energy from the external load, coming from the eccentric contraction-based accelerating movement, cannot “recoil” in the decelerating movement [19]. It has been proposed that the excess “unrecoiled” energy coming from accelerating/eccentric movements is partially absorbed by muscles and other tissues like connective tissue, extracellular matrix [26], and even peripheral proprioceptive nerve terminals [6] in a damaging way [19]. Correspondingly, Proske and Gandevia have proposed that damaging eccentric exercise is to blame for the impairment of proprioception [27]. Indeed, one symptom of DOMS is diminished proprioceptive function right after eccentric exercise, suggested to be derived from the muscle spindle [28].

The acute compression proprioceptive axonopathy theory of DOMS postulated that when the force production is unacceptably insufficient, then cognitive demand induced ASR, or “over-reaching”, could prevail as a driver of eccentric contractions [6]. From a neuro-energetics point of view, the extension of homeostasis is allostasis, meaning the maintenance of stability in an energy-demanding perturbed environment, like in severe physical challenge [29]. Accordingly, the acute state of allostatic stress is the equivalent of the proposed ASR time window. The sensory afferent terminals of the muscle spindle could be microdamaged by repetitive compressive eccentric contractions during this ASR time window [6] or during allostatic stress. Correspondingly, it is proposed that the inactivated Piezo2 ion channels at the Type Ia terminals could go through pathological mechano-energetic microdamage and as a result could become “leaky” during allostatic stress in DOMS [7,20,22]. Notably, the proposed type of sensory terminal lesion, coined as terminal arbor degeneration, evolves after a dose-limiting manner in an acute and chronic way as well, and not associated with Wallerian-like axonal degeneration [19,30].

In summary, DOMS is caused by unaccustomed or strenuous microdamaging contractions and, as a result, the proprioceptive terminals in the muscle spindle could go through a mechano-energetic impairment under an allostatic stress.

### 3.2. Dichotomous Injury Mechanism of DOMS

Hody et al. [13] divided DOMS into two phases, where the initial mechanical damage is followed by a more severe secondary damage, and referred to Morgan et al. [31], who described these phases as primary and secondary damage phases.

The acute compression proprioceptive axonopathy theory of DOMS also emphasized the bi-phasic nature of this non-contact injury mechanism (see Table 1) [6,7]. According to this theory, mechanical hyperalgesia could be initiated by an acute painless impairment of glutamate vesicular release and Piezo2 channelopathy (primary damage phase) enhanced by inflammatory damage and pain (secondary damage phase) [6,7,20,22]. C-fibers convey pain from microinjured extrafusal tissues in DOMS [4], but the theory proposed that the Piezo2 channelopathy of the microinjured Type Ia sensory terminals in the muscle spindle could exclusively provide the “open gate” to mechanical hyperalgesia in DOMS [6,7,20] by switching to hyperexcited Type II sensory neurons that are contributing to nociception and arriving earlier to the “open gate” under this acute pathological condition [6]. Therefore, the involvement of C-fibers in mechanical hyperalgesia is only a secondary, but essential contribution, providing the slow temporal summation of pain [32], part of the theory that the coupling of the pain pathways is facilitated by sympathetic nervous system (SNS) activity [33] once the gate is open [6]. It is noteworthy that “open gate” means the involvement of the central nervous system (CNS) [6].

The Type Ia proprioceptive fiber is proposed to be hyperexcited by glutamate intrafusally [19]. The COX-1–PGE2 pathway could hyperexcite Type II sensory fibers in the muscle spindle, based on the paper of Sun et al. [6,34]. Mizumura et al. [4] demonstrated that the COX-2–PGE2–GDNF pathway could hyperexcite Type III sensory fibers, while Murase et al. [35] showed that the COX-2–NGF pathway could hyperexcite Type IV sensory or C-fibers in the microdamaged extrafusal muscle.

Furthermore, Murase et al. [36] also demonstrated that there is a cross-talk on the level of COX-2 that could be one basis for the Type III and C-fiber coupling in the inflammatory pain pathway [6,19]. Moreover, the acute compression axonopathy theory of DOMS postulated that PGE2 could also provide the basis for cross-talk between the Type II fiber (neuropathic pathway) and the coupled Type III/IV sensory fibers [6,19]. The theory highlighted glutamate and the COX-1–PGE2 excitatory pathways [19] that could lead to pathological hyperexcitation, impairment of glutamate vesicular release, and Piezo2 channelopathy on Type Ia fibers in the primary damage phase of DOMS [7,20]. The consequence of this primary microdamage is suggested to be the compensatory switch of the static phase firing encoding from Type Ia fibers to Type II fibers [7,19] which could be the reason behind decreased proprioception and delayed medium latency response (MLR) of the stretch reflex right after fatiguing eccentric exercise [7,28].

The secondary damage phase is a harsher tissue damage, and it is the direct result of the primary damage phase, ending in impaired proprioception due to Piezo2 channelopathy [7,20,22].

In summary, the critical primary damage is suggested to be neural and within the intrafusal space, while the secondary damage takes place in the extrafusal space. The primary injury phase without the secondary injury phase is a painless microinjured condition without DOMS, proposed to last only 2–3 days [7,20]. The secondary damage without the primary injury could not cause DOMS either; however, it could come with exercise-induced muscle damage (EIMD) without delayed onset of soreness (see Table 1). EIMD arises when muscles are damaged after exercise, and is often induced after unaccustomed exercise, especially if it heavily involves eccentric contractions [37]. However, it should be emphasized that EIMD is not equivalent to DOMS. For instance, DOMS could evolve without muscle damage [38], due to vibration effect [39]. It is important to note that Piezo2 is contributing to vibration sensing [40].

**Table 1:** Microinjury mechanism in EIMD and DOMS *, adapted from exercise-induced microdamages [22].

	Exercise-Induced Muscle Damage (EIMD ^1^)		Delayed Onset Muscle Soreness (DOMS ^2^)	
**Primary injury phase**	NO	No intrafusal proprioceptive terminal microdamage and no intrafusal allostatic impairment of lactate shuttle	YES	Intrafusal proprioceptive terminal microdamage and intrafusal allostatic impairment of lactate shuttle
**Secondary injury phase**	YES	Extrafusal microdamage of muscle with C fiber contribution	YES	Extrafusal microdamage with C fiber contribution
**Soreness condition**	Exercise-induced soreness without delayed onset		DOMS lasting up to 7 days	

^1^ Exercise-induced muscle damage (EIMD): muscle damage evolves after exercise that is often unaccustomed, especially if it heavily involves eccentric contractions [38], and could be associated with soreness without delayed onset. ^2^ Delayed onset muscle damage (DOMS): often caused by unaccustomed and strenuous exercise entailing repetitive, fatiguing eccentric contractions, associated with delayed onset of soreness, muscle stiffness, swelling, loss of force-generating capacity, reduced joint range of motion, and diminished proprioceptive function [2] and could evolve without muscle damage [38]. * EIMD does not comprise proprioceptive microdamage within the muscle spindle, in contrast to DOMS. Both EIMD and DOMS contain extrafusal microdamage; however, in the case of DOMS muscle damage is not essential, e.g., extracellular matrix rupture could be enough. DOMS is theorized to evolve only if both intrafusal proprioceptive and extrafusal microdamages are present, involving Piezo2 channels [22].

## 4. Molecular Mechanism of Lactate’s Involvement in DOMS

Henceforward, the current opinion aims to highlight the contributory role of lactate metabolism in both damage phases of DOMS pathophysiology.

### 4.1. Lactate in the Primary Damage Phase of DOMS

#### 4.1.1. Impact of Lactate on Type Ia Fibers

Satellite cells are astrocyte-like stem cells, and they have a functional role in muscle plasticity. Furthermore, satellite cells may take part in the plasticity of peripheral neurons, as astrocytes do in the CNS [41]. It was also proposed that activated intrafusal satellite cells could contribute to the permeability increase of the selective barrier of the muscle spindle [19], as astrocytes do in the CNS [42]. It is noteworthy that satellite cells are activated under mechanical strain. In addition, satellite cells have a role in the metabolic switch that increases glycolysis [43], as astrocytes do [44,45]. Moreover, satellite cells also contribute to the maintenance of stability in neuromuscular junctions [46]. Certain studies could imply a similar function for intrafusal satellite cells in the intrafusal compartment, not only in the extrafusal one [19,47,48]. These supporting mechanisms of satellite cells are within homeostasis, or even the extension of homeostasis, when allostatic stress could prevail. However, under allostasis, we should specifically consider the neurocentric dimension, when neurons switch from glucose to lactate, as energy substrate [29]. Even taking a more complex approach, when intrafusal satellite cells co-exist with neurons metabolically, could provide us with better understanding. This type of metabolic machinery may prevail in the brain, according to the astrocyte-neuron lactate shuttle (ANLS) hypothesis [49]. Correspondingly, astrocytes are increasing their glucose uptake, glycolysis, and the lactate release in response to hyperexcited neurons that increase glutamatergic activity [29]. Hence, the glutamate transmitter pool could be replenished, and eventually astrocytes could release more lactate that is taken up by neurons as fuel [29].

An analogue metabolic machinery of ANLS should be considered in the muscle spindle in reference to Type Ia proprioceptive sensory neurons and intrafusal satellite cells. It has been hypothesized that under unaccustomed or strenuous repetitive contractions, a mechano-energetic microinjury could prevail on Type Ia peripheral terminals [6]. The mechano part of the microinjury is proposed to be a Piezo2 channelopathy [7,20,22], while the metabo-energetic part is the impairment of the glutamate vesicular release [7,20,22]. The latter could be due to impaired mitochondrial trafficking to the proprioceptive terminals and/or anaerobic metabolism of glucose with resultant increased lactate production, hence this metabolic switch could eventually lead to acidosis and excessive lactate as energy substrate for proprioceptive neurons that could eventually impair glutamate vesicular release. The consequence of this Type Ia terminal mechano-energetic microinjury was theorized to be the conduction velocity loss relative to Type II, meaning the “open gate” [6]. Furthermore, it was hypothesized that the static phase firing encoding of Type Ia fibers will be impaired due to this microinjury, namely Piezo2 channelopathy and impairment of glutamate vesicular release, and the static phase firing encoding would be conveyed by Type II fibers in this case [19,20,22]. This switch from primary monosynaptic pathway to the secondary polysynaptic compensatory pathway was proposed to delay the medium latency response of the stretch reflex [19,20,21,22]. Indeed, electrophysiological study showed that DOMS-inducing exercise significantly delayed the MLR [7].

In summary, an ANLS analogue machinery could exist in the muscle spindle and the allostatic impairment of this mechanism could contribute to the primary damage phase of DOMS, meaning that lactate directly or indirectly could contribute to the permeability increase of the muscle spindle capsule, lactate nourishment of intrafusal proprioceptive sensory nerves, and even to the impairment of glutamate vesicular release in a neural microinjured state. Notably, the theoretical permeability increase of the selective membrane function of the muscle spindle capsule could not be accidental, but an “open gate” to lactate nourishment for neurons under the strained intrafusal environment of DOMS. Moreover, the work of Wernbom et al. seems to substantiate the above theory; however, training under ischemic conditions is not the detailed subject of this paper [50]. It is also important to note that concentric exercise is accompanied by higher metabolism [1], but most likely not intrafusally [19]. Therefore, the current author proposes that concentric exercise does not cause DOMS due to the diminished intrafusal muscle and intrafusal proprioceptive static phase firing loading, not to mention the lower intrafusal metabolism.

#### 4.1.2. Impact of Lactate on Type II Fibers

The metabolic switch to anaerobic metabolism of glucose in an allostatic stress environment could lead to acidosis and excessive lactate production, and that could inhibit astrocytic glutamate uptake, hence contributing to excitatory neuronal injury in the CNS [51]. An analogous glutamate vesicular release impairment could lead to the proposed excitatory Type Ia terminal microinjury in the primary damage phase of DOMS [7,20,22]. Therefore, the impairment of metabolic switch to adequate neuronal lactate nourishment leads to acidosis and excessive lactate production in an acute allostatic strained environment. The selective barrier permeability increase induced by intrafusal satellite cells could pave the way for additional excessive acidosis and lactate influx from extrafusal space [19]. Tissue acidosis-induced activation of acid-sensing ion channels (ASICs), a group of amiloride-sensitive ligand-gated ion channels, could critically contribute to neuronal injury, both in the CNS and the peripheral nervous system [52]. The activation of ASIC3 ion channels in Type II sensory neurons in the muscle spindle could be a good candidate for this mechanism, since ASIC3 known to have a role in mechanotransduction in proprioceptors [53]. Furthermore, ASIC3 is implicated in nociception [54], as the acute compression axonopathy theory proposes [55]. Correspondingly, the acute compression axonopathy theory of DOMS also suggested that the COX-1–PGE2 pathway could hyperexcite Type II sensory fibers [6,19] and it has been shown that PGE2 is involved in hyperalgesia by amplifying ASICs in a dose-dependent way in primary sensory neurons [56]. It is important to note that ASIC3 currents are known to be enhanced by lactate [57].

### 4.2. Lactate in the Secondary Damage Phase of DOMS

Lactate might have a role in satellite cell activation [58]. Satellite cells produce NGF and GDNF that are crucial players of DOMS [4]. It is also known that bradykinin and its agonist have a role in the breakdown of selective barrier permeability through B2 bradykinin receptors [59,60,61]. The findings, that the elevation of pain sensitivity is greater for fascia than for muscle [62,63,64], could imply that bradykinin has a role in increasing the selective barrier permeability of fascia. Indeed, Murase et al. also demonstrated that bradykinin plays an essential role in the mechanical hyperalgesia of DOMS after eccentric exercise [35], which is certainly the secondary damage phase of DOMS. Notably, lactate is known to potentiate the effect of bradykinin on neurons [65]; therefore, it has a role even in the initiation of the secondary damage phase of DOMS. Léger et al. rightly concluded that lactic acid may contribute to acute muscle soreness after intense exercise, due to fatigue [14], which is also the case in the absence of intrafusal proprioceptive microinjury.

Notably, ASICs are activated in the extrafusal small sensory fibers as dual function proteins for chemo- and mechano-sensing and have a role in inflammatory muscle pain in the secondary damage phase of DOMS [66]. Other ion channels, like TRP1 and TRPV4, are also pivotal contributors to this secondary damage phase of DOMS [67]. Moreover, the excitatory COX-2–NGF pathway on Type IV fibers [35] could crosstalk with the NGF-TrkA-Piezo2 signaling axis on Type III sensory afferent neurons in noxious mechanical stimulation [68,69], thereby also coupling the Type III/IV fibers, as is suggested by the acute compression axonopathy theory of DOMS [6,22]. It is again important to note that eccentric exercise activates B2 bradykinin receptors and NGF [4], while lactate potentiates bradykinin’s effect on neurons [65].

In summary, lactate may even have a role in the commencement of the secondary damage phase of DOMS, most importantly by potentiating the effect of bradykinin.

## 5. Conclusions

Altogether, lactate seems to have an essential role in the initiation of the primary damage phase of delayed onset muscle soreness in the Type Ia sensory terminals within the intrafusal space. In addition, lactate may also contribute to the secondary damage phase of delayed onset muscle soreness in the extrafusal space, primarily by potentiating the role of bradykinin. Unaccustomed or strenuous eccentric contractions are suggested to facilitate more lactate nourishment of hyperexcited proprioceptive sensory neurons in the muscle spindle; however, excessive acidosis and lactate could contribute to impaired proprioception and increased nociception under the proposed pathological condition. After all, the critical role of lactate in the metabolism of proprioceptive sensory neurons should not be void, if a neurocentric view is taken. The neurocentric view highlights the theory that proprioceptive sensory microdamage could be the pivotal mechanism in delayed onset muscle soreness pathophysiology. Emerging transcriptomic and proteomic analysis is one promising approach for enlightening the precise molecular characterization of muscle spindles [70], paving the way for future functional studies of the intrafusal proprioceptive terminals.
